# Bacteraemia and fungaemia in cystic fibrosis patients with febrile pulmonary exacerbation: a prospective observational study

**DOI:** 10.1186/s12890-017-0440-4

**Published:** 2017-06-29

**Authors:** Joerg Grosse-Onnebrink, Florian Stehling, Eva Tschiedel, Margarete Olivier, Uwe Mellies, Rene Schmidt, Jan Buer, Peter-Micheal Rath, Joerg Steinmann

**Affiliations:** 1 Department of Paediatric Respiratory Medicine, Cystic Fibrosis Centre, University Children’s Hospital Essen, Essen, Germany; 2 Institute of Biostatistics and Clinical Research, University of Muenster, Muenster, Germany; 3 Institute of Medical Microbiology, University Hospital Essen, Essen, Germany; 4 Present address: Department of General Paediatrics, Paediatric Pulmonology Unit, University Hospital Muenster, Albert-Schweitzer-Strasse 1, Building A1 D-, 48149 Muenster, Germany

## Abstract

**Background:**

Bloodstream pathogens can be identified by multiplex PCR (SeptiFast (SF)) or blood culture (BC); whether these pathogens are present in cystic fibrosis (CF) patients during febrile pulmonary exacerbations (FPE) has not been sufficiently studied.

**Methods:**

In this prospective observational study, blood from CF patients experiencing FPE was tested with SF and BC before the initiation of antibiotic treatment.

**Results:**

After contaminants had been excluded, 9 of 72 blood samples tested positive by BC or SF. SF exclusively detected four pathogens; BC, one. Pulmonary pathogen transmission was likely in all cases except for 2 cases of candidaemia, which were believed to be caused by catheter-related infections. For three cases, test results caused us to change the antibiotic regimen. Sensitivity (85.7% vs. 42.9%) and negative predictive value (98.4% vs. 87.0%) tended to be higher for SF than for BC.

**Conclusions:**

The results of SF and BC show that bacteraemia and fungaemia are present in CF patients during FPE and may affect antibiotic therapy. SF can help rule out catheter-related bloodstream infections.

## Background

The course of cystic fibrosis (CF) lung disease is characterized by repetitive episodes of bacterial, fungal, and viral airway infections that have a substantial impact on the progression of lung disease and on the patient’s quality of life. These intermittent episodes are acute worsening clinical symptoms, summarized as infective pulmonary exacerbations. It has not yet been determined whether pulmonary infective exacerbations among CF patients are associated with an increased risk of unrecognized bacteraemia or fungaemia and, additionally, whether microorganisms that colonize the respiratory tract of CF patients may cause bloodstream infections.

In addition, it is still unknown whether collecting blood cultures (BCs) has any benefit in these situations. If bacteraemia or fungaemia is present during febrile pulmonary exacerbations (FPE) in CF patients, knowledge about possible microorganisms in the blood can be essential for ensuring that life-threatening bloodstream infections are not overlooked. Thus, it is of interest whether FPE increase the incidence of bacteraemia or fungaemia; if they do, more aggressive and urgent treatment with antibiotics may be indicated for CF patients.

In addition to BCs, polymerase chain reaction (PCR)-based methods have been introduced as an additional diagnostic tool for detecting blood stream microorganisms. SeptiFast (SF) real-time PCR is a polymerase chain–based test for detecting the DNA of 25 pathogenic microorganisms in the blood [1]. SF is used to detect bloodstream infections in patients with suspected sepsis [2, 3] and can be used in addition to BC [4]. Until now, the SF assay has not been used for CF patients.

To our knowledge, only one published study [5] has examined the incidence of positive findings from BCs collected during FPE among CF patients; that incidence was reported to be 3.5%. The study was limited by its small study population (18 patients with FPE) and the availability of BC alone for assessing bacteraemia or fungaemia.

We conducted this prospective observational study to test the hypotheses that bacteraemia or fungaemia is present during FPE among CF patients and that SF is more sensitive than BCs in detecting bloodstream infections.

## Methods

### Study site, patients, inclusion criteria

Patients for this prospective observational single-centre study were recruited from the Cystic Fibrosis Centre of the University Children’s Hospital Essen in Essen, Germany, between July 2009 and July 2011. The study was approved by the local ethics committee, and all patients, their parents, or both gave informed consent. Patients were enrolled in the study if they had CF, as diagnosed either by positive results from two sweat chloride tests or by detection of two mutations that are considered to cause CF; if they had a fever (i.e., body temperature ≥38.5 °C); and if they were hospitalized for an infective pulmonary exacerbation. Infective pulmonary exacerbation was defined by a modification of the Ramsey criteria [6]. Patients’ charts were reviewed for previous antimicrobial treatment or prednisolone administration.

### Blood cultures

After disinfection of the skin and the connector, 8 to 10 mL of blood (one aerobic and one anaerobic BC bottle) was collected by sterile venepuncture or from a central venous catheter, if one was present. Blood was inoculated in BC bottles and incubated for up to 7 days at 36 °C (BACTEC 9240 system; Becton-Dickinson, Heidelberg, Germany). If BC results were positive, aliquots were drawn for Gram staining and culturing on solid media. Microorganisms were identified with VITEK MS mass spectrometry (bioMerieux, Nürtingen, Germany) or the MicroScan Walk-Away system (Beckmann Coulter, Krefeld, Germany).

### SeptiFast

Whole blood for PCR analysis was drawn at the same time as blood was drawn for BCs. The LightCycler SeptiFast test (Roche Diagnostics, Mannheim, Germany) detects DNA of bacterial and fungal pathogens in 1.5 mL EDTA blood. The manufacturer states that SF can detect the following spectrum of species: *Escherichia coli, Klebsiella pneumonia/oxytoca, Serratia marcescens, Enterobacter cloacae/aerogenes, Proteus mirabilis, Pseudomonas aeruginosa, Acinetobacter baumannii,* coagulase-negative *Staphylococcus species, Staphylococcus aureus, Streptococcus pneumoniae, Streptococcus spp., Enterococcus faecium and E. faecalis, Candida albicans, C. tropicalis, C. parapsilosis, C. krusei, C. glabrata, and Aspergillus fumigatus.* The analytical sensitivity of the assay ranges from 3 to 100 colony-forming units (CFU)/mL, depending on the microorganism. The assay was performed according to the manufacturer’s instructions. After DNA had been extracted and amplified, PCR products were simultaneously detected by fluorescence and melting temperature analysis using specific hybridization probes and the SF identification software.

The identification of BC or SF contamination was based on previously published criteria [7–9], including the probability that the organism was a skin contaminant, other concurrent microbiology results, and clinical compatibility. We defined a contamination when microorganisms from the skin flora were detected in either BC or SF and if the clinical situation stabilized without modification of antibiotic therapy or removal of central venous catheters before culture results arrived.

### Statistical analysis

Basic clinical data are summarized in Table 1. Unless otherwise specified, the description of clinical data is based on the outcome at baseline examination (first episode of FPE). Distribution of metric variables is reported as median and interquartile range (IQR). Categorical data are described by absolute and relative frequencies. Lung function parameters are presented as absolute values and percent predicted values as calculated with the equations of Zapletal [10].

**Table 1:** Baseline characteristics of study participants

Baseline characteristics	Study participants (*n* = 28)
Age in years, median (IQR)	21.0 (16.0; 23.0)
Female, *n* (%)	11 (39.3)
*Pseudomonas aeruginosa* positive^a^, *n* (%)	15 (53.6)
*Staphylococcus aureus* (MSSA) positive^b^, *n* (%)	16 (57.1)
*Staphylococcus aureus* (MRSA) positive^b^, *n*	0
*Stenotrophomonas maltophilia* positive^b^, *n* (%)	1 (3.6)
*Burkholderia cepacia* positive^b^, *n* (%)	1 (3.6)
Body mass index, median (IQR)	18.3 (16.2; 19.6)
Pancreatic insufficient, *n* (%)	26 (92.9)
Diabetes mellitus, *n* (%)	10 (35.7)
ABPA, *n* (%)	1 (3.6)
ΔF508/ΔF508, *n* (%)	17 (60.7)
ΔF508 compound heterozygote, *n* (%)	7 (25.0)
Others^c^, *n* (%)	4 (14.3)
FVC, litres, median (IQR)	2.5 (1.6; 2.8)
FVC % predicted, median (IQR)	66.0 (47.8; 73.6)
FEV1, litres, median (IQR)	1.4 (0.9; 1.9)
FEV1 % predicted, median (IQR)	46.0 (43.0; 58.0)

*IQR* interquartile range, *MSSA*, methicillin-susceptible *S. aureus*, *MRSA* methicillin-resistant *S. aureus*, *ABPA* allergic bronchopulmonary aspergillosis, *FVC* forced vital capacity, *FEV* forced expiratory volume in 1 second

^a^ *Pseudomonas aeruginosa* positive is defined as two or more positive airway cultures in the previous year, currently receiving inhaled pseudomonal treatment, or both

^b^Positive is defined as two or more positive airway cultures in the previous year

^c^Others is defined as mutations known to cause cystic fibrosis other than ΔF508 or not identified

The primary outcome measure was the number of positive diagnostic test results obtained by SF and BCs (positive or negative findings for microorganisms). Bacteraemia or fungaemia was considered to be present (gold standard) if contamination of the probe was considered unlikely and was otherwise not present [11–13]. Data were recorded for all episodes of FPE that occurred among the patient cohort during the observational period of this study; thus, multiple observations were made per patient from subsequent episodes, resulting in clustered data. The frequency of episodes of FPE in association with positive or negative findings from SF or BC is displayed in Table 2.

**Table 2:** Frequency of positive or negative findings from blood cultures or SeptiFast polymerase chain reaction analyses of blood samples from cystic fibrosis patients experiencing episodes of febrile infective exacerbation

	BC+	BC-	total	Contaminant
SF+	2	6	8	2 (SF+ and BC-)
SF-	3	61	64	
total	5	67	72	
Contaminant	2 (BC+ and SF-)			

BC+, probe testing positive by blood culture

BC-, probe testing negative by blood culture

SF+, probe testing positive by SeptiFast polymerase chain reaction analysis

SF-, probe testing negative by SeptiFast polymerase chain reaction analysis Contaminant, probe considered to be a contaminant

The primary objective was assessment of sensitivity, specificity, positive predictive value (PPV), negative predictive value (NPV), and rate of positivity (ROP) of SF and BC as related to the gold standard. To account for clustering of data, we used the generalized estimation equation (GEE) approach [14, 15] with logit link function and exchangeable working correlation structure to calculate population-averaged sensitivity, specificity, PPV, NPV, and ROP estimates adjusted for correlation within patients. Estimates with 95% confidence interval (95% CI) are presented in Table 3. To compare sensitivity, specificity, ROP, PPV, and NPV of diagnostic tests (SF vs. BC) while accounting for clustering of data, we used an analogue of McNemar’s test based on GEE [16]. Corresponding two-tailed *P* values are given in Table 3. Univariable associations between SF and BC results and clinical data were analysed with the GEE approach with logit link function and exchangeable working correlation structure. The exchangeable working correlation structure was chosen for all GEE models because the time order of episodes of FPE is incidental.

**Table 3:** Measures describing how well blood cultures and SeptiFast polymerase chain reaction analyses capture the true presence or absence of disease, as related to gold standard, with 95% confidence interval

	Test		
% (95% CI)	SF	BC	*P* value
ROP	13.2% (6.7%–17.8%)	7.3% (2.8%–17.8%)	0.322
Sensitivity	85.7% (48.6%–97.5%)	42.9% (10.2%–83.2%)	0.250
Specificity	96.9% (87.9%–99.3%)	96.9% (88.6%–99.2%)	1.000
PPV	74.7% (36.0%–93.9%)	60.2% (18.5%–90.1%)	0.255
NPV	98.4% (89.3%–99.8%)	87.0% (86.2%–94.1%)	0.105

*SF* SeptiFast polymerase chain reaction analysis, *BC* blood culture, *ROP* rate of positivity, *PPV* positive predictive value, *NPV* negative predictive value

*P* value: Two-sided *P* value of the null hypothesis of equal ROP, sensitivity, specificity, PPV, and NPV of SF and BC

Sensitivity: P (Test = positive | gold standard = positive)

Specificity: P (Test = negative | gold standard = negative)

PPV: P (gold standard = positive | Test = positive)

NPV: P (gold standard = negative | Test = negative)

ROP: P (Test = positive)

Analyses were performed with SPSS (version 23; SPSS Inc., Chicago, IL, USA) and were regarded as explorative with *P* values displayed for descriptive reasons so that we could detect and study meaningful effects. In particular, no adjustment for multiple testing was performed. “Significance” refers to local statistical significance, defined as a local, unadjusted *P* value lower than 0.05.

## Results

Results of analyses of paired blood samples tested by BC and SF for 72 episodes of FPE are shown in Tables 2 and 4. Four samples were considered to be contaminants: two samples of *E. cloacae* with positive results by SF only, and two samples of *S. epidermidis* with positive results by BC only. Excluding contaminants, either BC or SF detected a pathogen in 7 of 72 samples (9.7%), and both methods detected pathogens in 2 samples. Excluding contaminants, 3 of 72 samples tested positive by BC (4.2%), and 6 of 72 samples (8.3%) tested positive by SF.

**Table 4:** Pathogens detected by blood culture or by SeptiFast polymerase chain reaction analysis, and their clinical relevance

Pathogen	SF	BC	Probably contaminant	Pathogen in sputum^a^	Therapeutic intervention	Comment
*C. albicans*	+	+	-	+	Fluconazole i.v.	Totally implantable venous access port; sepsis
*C. albicans*	+	+	-	+	Fluconazole i.v.	Central venous line; candida culture at top of line positive; sepsis
*S. maltophilia*	+	-	-	+	Trimethoprim/sulfamethoxazole i.v.	Intermittent respiratory tract colonization with *S. maltophilia*
*P. aeruginosa*	+	-	-	+	-	Intermittent respiratory tract colonization with *P. aeruginosa*; *P. aeruginosa* antibodies negative; empiric antibiotic treatment covered *P. aeruginosa*
*P. aeruginosa*	+	-	-	+	-	Chronic respiratory tract infection with *P. aeruginosa;* empiric antibiotic treatment covered *P. aeruginosa*
*K. pneumoniae*	+	-	-	-	-	Empiric antibiotic treatment covered *K. pneumonia*
*S. aureus*	-	+	-	Intermittent	-	Erysipelas, left foot; Empiric treatment covered *S. aureus*
*E. cloacae*	+	-	+	-	-	
*E. cloacae*	+	-	+	-	-	
*S. epidermidis*	-	+	+	-	-	
*S. epidermidis*	-	+	+	-	-	

*SF* SeptiFast, *BC* blood culture, *C* Candida, *P* Pseudomonas, *K* Klebsiella, *E* Enterobacter, *S* Staphylococcus, *i.v* intravenous

^a^Pathogen in sputum: pathogen detected in sputum during the previous year

With BC, ROP was 7.3%, sensitivity was 42.9%, specificity was 96.9%, PPV was 60.2%, and NPV was 87.0%. With SF, ROP (13.2%, *P* = 0.322), sensitivity (85.7%, *P* = 0.250), PPV (74.7%, *P* = 0.255), and NPV (98.4%, *P* = 0.105) tended to be higher than with BC. Specificities for SF (96.9%) and BC (96.9%) were similar (*P* = 1.0) (Table 3).

SF detected four pathogens that were not detected by BC, whereas BC detected one pathogen that was not detected by SF (Table 2). After exclusion of contaminants, *S. maltophilia*, *P. aeruginosa* (twice) and *K. pneumoniae* pathogens were exclusively detected by SF. *S. aureus* was detected exclusively by BC. Despite one case of *K. pneumoniae,* pathogens detected by SF or BC were regularly detected in sputum samples from the same patients either during the infective exacerbation period or within the year prior to the infective exacerbation period or both.

Because antimicrobial treatment may substantially affect the sensitivity of BC, patients’ charts were examined for documentation of previous antimicrobial treatment. Eight samples came from patients who had been treated with azithromycin three times per week (dosage depending on bodyweight: 250 mg for patients weighing <40 kg; 500 mg for patients weighing ≥40 kg) before infective exacerbation. Preexisting antibiotic treatment did not correlate with positive results on SF (*P* = 0.976) or BC (*P* = 0.512). During 10 of 72 FPE episodes, patients had been treated with prednisolone (dose, 5 or 10 mg), but none of the samples taken during these episodes tested positive with either SF or BC. All samples that tested positive for *C. albicans* with either BC or SF had been collected from a patient with a central venous catheter. Positive SF findings changed the therapeutic strategy in three cases: in two cases fluconazole was added after SF detected *C. albicans*, and in one case trimethoprim/sulfamethoxazole was added after SF detected *S. maltophilia* (Table 4).

## Discussion

This is the first study to detect microorganisms in blood with both BC and SF during FPE in CF patients. After contaminants had been excluded, 9 SF or BC probes tested positive during 72 episodes of FPE. Reports of bloodstream infections among CF patients are rare: in a retrospective study of Cargill et al. [17] blood stream infections in CF patients were analysed but the relation of FPE and blood stream infections was not systematically evaluated and recently a retrospective study primarily addressing the pattern of blood stream infection in adult CF patients has been published [18]. The only prospective study that has systematically evaluated the incidence of bloodstream infections during infective exacerbations in CF patients was published 25 years ago by Fahy et al. [5]. PCR assays for detecting the DNA of microorganisms in blood have not previously been used for CF patients. The study by Fahy et al. [5] found that 3 of 89 samples collected during FPE tested positive with BC (prevalence, 0.035). In our study, 3 of 72 samples collected during FPE tested positive with BC (prevalence, 0.042). Thus, the results of our study confirm the results of the study by Fahy et al.

SF detected 4 pathogens in blood that were not detected by BC. The prevalence of samples that tested positive with either BC or SF was 7 of 72 (0.097), which is more than twice the prevalence of positive findings than with BC alone. Because, in contrast to BC, SF is a PCR-based test for detecting pathogen DNA, the vitality of pathogens in blood can be questioned. It is well known that studies involving subjects with systemic inflammatory response syndrome (SIRS) or sepsis have shown that SF can detect pathogens in blood that cannot be detected by BC and that positive SF results in these patients are positively correlated with disease severity [12, 19]. In our study the clinical course of patients with positive SF results supports the presence of an infection by the detected pathogens.

All pathogens that were found by SF and BC, except for contaminants and *K. pneumoniae*, had been detected in sputum samples from the same patients during the previous year. *K. pneumoniae* was detected by SF alone in one patient, and it had already been covered by empiric antibiotic therapy. *K. pneumoniae* had not previously been found in a sputum culture or by BC in this patient. In one sample, *S. aureus* was detected by BC only. The patient had erysipelas, and his airways were chronically colonized with *S. aureus*. Whether the site of entry was the lung or the tissue affected by erysipelas could not be determined. In one case, SF but not BC detected *S. maltophilia*. The *S. maltophilia* antibody titre was high, 1:2560, a finding indicating chronic colonization or infection [20]; the bacterium had been detected in sputum cultures from this patient during the previous year. These findings suggest that *S. maltophilia* may have contributed to the FPE. Therefore, we added trimethoprim/sulfamethoxazole to the empirically selected antibiotic therapy. The findings of our study support the hypothesis that, in CF patients, pathogens are transmitted by the airways into the blood during FPE.

In two cases, *C. albicans* was detected by both SF and BC. In both of these cases, a central venous line was present; central venous lines are a known risk factor for candidaemia [21]. The clinical course of these patients was characterized by high fever and a severely impaired condition but no signs of septic shock; these findings were in line with the symptoms of candidaemia. For one of these patients, the results of SF changed the treatment strategy: fluconazole was added to the treatment regimen. For the other patient, the BC results confirmed the SF results, what caused us to add fluconazole to the treatment regimen. Thus, SF can help with the initiation of appropriate and early treatment for CF patients, especially if the intravenous antimicrobial therapy is intended as at-home treatment during FPE. The high NPV of SF for detecting pathogens in the blood (98.4%) and the rapid availability of the test may be beneficial, because negative SF results can support a prompt decision if at-home intravenous treatment with antibiotics is considered appropriate for CF patients with a central venous line.

The ROP, sensitivity, PPV, and NPV for SF tended to be higher than those for BC. The sensitivity of SF (85.7%) was higher than that of BC, and the specificity of SF (96.9%) was comparable to that found by meta-analyses involving heterogeneous groups of patients, most of whom did not have CF (Chang et al.: sensitivity, 0.75; 95% CI, 0.65–0.83; specificity, 0.92; 95% CI, 0.90–0.95; Dark et al.: sensitivity, 0.68; 95% CI, 0.63–0.73; specificity, 0.86; 95% CI, 0.84–0.89) [2, 3]. Our findings confirmed the results of these meta-analyses for patients with presumed SIRS or sepsis in CF patients with FPE. Although sensitivity, specificity, PPV and NPV for SF tended to be higher or were equal to BC, SF cannot replace BC testing because the spectrum of detectable pathogens in SF testing is limited.

In patients with suspected sepsis who received antibiotics prior to BC and SF, SF is considered to be more sensitive compared to BC in detecting pathogens (see Pasqualini et al. [22]). We analysed the patient’s records regarding pre-treatment with antibiotics. In this group one patient had positive results for both SF and BC (*C. albicans*). Thus, we cannot draw conclusions regarding differences in sensitivity between SF and BC in CF patients with antibiotic treatment prior to FPE.

The prevalence of positive results with SF (0.083) in our cohort was in the lower part of the prevalence range of patients with suspected sepsis (0.07 to 0.85) and lower than that of immunocompromised patients (0.25 to 0.41) [2]. Although a growing body of evidence suggests that the immune defence mechanisms of CF patients are altered [23–30], our study does not support the hypothesis that this alteration leads to an increased number of episodes of bacteraemia and fungaemia among these patients, a finding that is in line with clinical observations.

Our study is limited by the relatively small number of CF patients with FPE. Therefore, we could not draw conclusions about the clinical usefulness of SF or about a cost-benefit calculation. Our study does not allow conclusions about whether empiric antimicrobial therapy for CF exacerbations may be improved by regular SF or BC testing. Larger prospective trials with a control group are necessary for answering this question. The aim of our study was not to determine whether bloodstream pathogens might be present in CF patients with non-febrile pulmonary infective exacerbations; the study included only febrile patients, because in these patients the overlap with sepsis or SIRS criteria is more pronounced. A strength of our study is its prospective and standardised study design.

## Conclusions

The results of our study indicate that bacteraemia and fungaemia are present in CF patients with FPE but occur less frequently in these patients than in patients with suspected SIRS or sepsis. It is likely that for CF patients the route of transmission for bloodstream infections with bacteria or fungi is via the lung or via central venous catheters. Among CF patients with a central venous catheter, a catheter-related bloodstream infection can be misinterpreted as a FPE. SF can help rule out catheter-related infections, a function that is of particular interest to patients scheduled for at-home antibiotic intravenous treatment.

## Abbreviations

95% CI: 95% confidence interval
BC: Blood culture
CF: Cystic fibrosis
FPE: Febrile pulmonary exacerbations
GEE: Generalized estimation equation
NPV: Negative predictive value
PCR: Polymerase chain reaction
PPV: Positive predictive value
ROP: Rate of positivity
SF: SeptiFast
SIRS: Systemic inflammatory response syndrome

## References

[1] Lehmann LE, Hunfeld KP, Emrich T, Haberhausen G, Wissing H, Hoeft A, Stuber F (2008). A multiplex real-time PCR assay for rapid detection and differentiation of 25 bacterial and fungal pathogens from whole blood samples. Med Microbiol Immunol.

[2] Chang SS, Hsieh WH, Liu TS, Lee SH, Wang CH, Chou HC, Yeo YH, Tseng CP, Lee CC (2013). Multiplex PCR system for rapid detection of pathogens in patients with presumed sepsis - a systemic review and meta-analysis. PLoS One.

[3] Dark P, Blackwood B, Gates S, McAuley D, Perkins GD, McMullan R, Wilson C, Graham D, Timms K, Warhurst G (2015). Accuracy of LightCycler((R)) SeptiFast for the detection and identification of pathogens in the blood of patients with suspected sepsis: a systematic review and meta-analysis. Intensive Care Med.

[4] Rath PM, Saner F, Paul A, Lehmann N, Steinmann E, Buer J, Steinmann J (2012). Multiplex PCR for rapid and improved diagnosis of bloodstream infections in liver transplant recipients. J Clin Microbiol.

[5] Fahy JV, Keoghan MT, Crummy EJ, FitzGerald MX (1991). Bacteraemia and fungaemia in adults with cystic fibrosis. J Infect.

[6] Ramsey BW, Pepe MS, Quan JM, Otto KL, Montgomery AB, Williams-Warren J, Vasiljev KM, Borowitz D, Bowman CM, Marshall BC (1999). Intermittent administration of inhaled tobramycin in patients with cystic fibrosis. Cystic Fibrosis Inhaled Tobramycin Study Group. N Engl J Med.

[7] Everts RJ, Vinson EN, Adholla PO, Reller LB (2001). Contamination of catheter-drawn blood cultures. J Clin Microbiol.

[8] Hall KK, Lyman JA (2006). Updated review of blood culture contamination. Clin Microbiol Rev.

[9] Weinstein MP, Towns ML, Quartey SM, Mirrett S, Reimer LG, Parmigiani G, Reller LB (1997). The clinical significance of positive blood cultures in the 1990s: a prospective comprehensive evaluation of the microbiology, epidemiology, and outcome of bacteremia and fungemia in adults. Clin. Infect. Dis.: an official publication of the Infectious Diseases Society of America.

[10] Zapletal A, Paul T, Samanek M (1977). Significance of contemporary methods of lung function testing for the detection of airway obstruction in children and adolescents (author's transl). Z Erkr Atmungsorgane.

[11] Lehmann LE, Hunfeld KP, Steinbrucker M, Brade V, Book M, Seifert H, Bingold T, Hoeft A, Wissing H, Stuber F (2010). Improved detection of blood stream pathogens by real-time PCR in severe sepsis. Intensive Care Med.

[12] Bloos F, Hinder F, Becker K, Sachse S, Mekontso Dessap A, Straube E, Cattoir V, Brun-Buisson C, Reinhart K, Peters G (2010). A multicenter trial to compare blood culture with polymerase chain reaction in severe human sepsis. Intensive Care Med.

[13] Westh H, Lisby G, Breysse F, Boddinghaus B, Chomarat M, Gant V, Goglio A, Raglio A, Schuster H, Stuber F (2009). Multiplex real-time PCR and blood culture for identification of bloodstream pathogens in patients with suspected sepsis. Clin. Microbiol. Infect..

[14] Zeger SL, Liang KY (1986). Longitudinal Data-Analysis for Discrete and Continuous Outcomes. Biometrics.

[15] Williams RL (2000). A note on robust variance estimation for cluster-correlated data. Biometrics.

[16] Leisenring W, Alonzo T, Pepe MS (2000). Comparisons of predictive values of binary medical diagnostic tests for paired designs. Biometrics.

[17] Cargill J, Etherington C, Peckham D, Conway S, Denton M (2012). Bloodstream infections in cystic fibrosis: nine years of experience in both adults and children. J. Cyst. Fibros.: official journal of the European Cystic Fibrosis Society.

[18] Vender RJ, Vender RL (2016). Clinical Impact of Blood Culture Results in Acutely Ill Hospitalized Adult Patients With Cystic Fibrosis. J.Clin. Med. Res..

[19] Bauer S, Kirschning CJ, Hacker H, Redecke V, Hausmann S, Akira S, Wagner H, Lipford GB (2001). Human TLR9 confers responsiveness to bacterial DNA via species-specific CpG motif recognition. Proc Natl Acad Sci U S A.

[20] Goncalves Vidigal P, Schmidt D, Stehling F, Mellies U, Steinmann E, Buer J, Rath PM, Steinmann J (2013). Development of a quantitative immunofluorescence assay for detection of Stenotrophomonas maltophilia antibodies in patients with cystic fibrosis. J. Cyst. Fibros.: official journal of the European Cystic Fibrosis Society.

[21] Bassetti M, Merelli M, Ansaldi F, de Florentiis D, Sartor A, Scarparo C, Callegari A, Righi E (2015). Clinical and therapeutic aspects of candidemia: a five year single centre study. PLoS One.

[22] Pasqualini L, Mencacci A, Leli C, Montagna P, Cardaccia A, Cenci E, Montecarlo I, Pirro M, di Filippo F, Cistaro E (2012). Diagnostic performance of a multiple real-time PCR assay in patients with suspected sepsis hospitalized in an internal medicine ward. J Clin Microbiol.

[23] Painter RG, Marrero L, Lombard GA, Valentine VG, Nauseef WM, Wang G (2010). CFTR-mediated halide transport in phagosomes of human neutrophils. J Leukoc Biol.

[24] Painter RG, Valentine VG, Lanson NA, Leidal K, Zhang Q, Lombard G, Thompson C, Viswanathan A, Nauseef WM, Wang G (2006). CFTR Expression in human neutrophils and the phagolysosomal chlorination defect in cystic fibrosis. Biochemistry.

[25] Painter RG, Bonvillain RW, Valentine VG, Lombard GA, LaPlace SG, Nauseef WM, Wang G (2008). The role of chloride anion and CFTR in killing of Pseudomonas aeruginosa by normal and CF neutrophils. J Leukoc Biol.

[26] Bonvillain RW, Painter RG, Adams DE, Viswanathan A, Lanson NA, Wang G (2010). RNA interference against CFTR affects HL60-derived neutrophil microbicidal function. Free Radic Biol Med.

[27] Phennicie RT, Sullivan MJ, Singer JT, Yoder JA, Kim CH (2010). Specific resistance to Pseudomonas aeruginosa infection in zebrafish is mediated by the cystic fibrosis transmembrane conductance regulator. Infect Immun.

[28] Zhou Y, Song K, Painter RG, Aiken M, Reiser J, Stanton BA, Nauseef WM, Wang G (2013). Cystic fibrosis transmembrane conductance regulator recruitment to phagosomes in neutrophils. J. Innate Immun..

[29] Rieber N, Hector A, Carevic M, Hartl D (2014). Current concepts of immune dysregulation in cystic fibrosis. Int J Biochem Cell Biol.

[30] Hartl D, Gaggar A, Bruscia E, Hector A, Marcos V, Jung A, Greene C, McElvaney G, Mall M, Doring G (2012). Innate immunity in cystic fibrosis lung disease. J. Cyst. Fibros.: official journal of the European Cystic Fibrosis Society.

